# Consistency and sources of divergence in recommendations on screening with questionnaires for presently experienced health problems or symptoms: a comparison of recommendations from the Canadian Task Force on Preventive Health Care, UK National Screening Committee, and US Preventive Services Task Force

**DOI:** 10.1186/s12916-017-0903-8

**Published:** 2017-08-09

**Authors:** Brett D. Thombs, Nazanin Saadat, Kira E. Riehm, Justin Michael Karter, Akansha Vaswani, Bonnie K. Andrews, Peter Simons, Lisa Cosgrove

**Affiliations:** 1 Department of Psychiatry, McGill University, Montréal, Québec Canada; 2 Department of Epidemiology, Biostatistics, and Occupational Health, McGill University, Montréal, Québec Canada; 3 Department of Medicine, McGill University, Montréal, Québec Canada; 4 Department of Psychology, McGill University, Montréal, Québec Canada; 5 Department of Educational and Counselling Psychology, McGill University, Montréal, Québec Canada; 6 Lady Davis Institute for Medical Research, Jewish General Hospital, Montréal, Québec Canada; 7 Department of Counseling and School Psychology, University of Massachusetts Boston, Boston, Massachusetts USA

**Keywords:** Screening, Self-report questionnaires, Preventive healthcare, Healthcare guidelines

## Abstract

**Background:**

Recently, health screening recommendations have gone beyond screening for early-stage, asymptomatic disease to include “screening” for presently experienced health problems and symptoms using self-report questionnaires. We examined recommendations from three major national guideline organizations to determine the consistency of recommendations, identify sources of divergent recommendations, and determine if guideline organizations have identified any direct randomized controlled trial (RCT) evidence for the effectiveness of questionnaire-based screening.

**Methods:**

We reviewed recommendation statements listed by the Canadian Task Force on Preventive Health Care (CTFPHC), the United Kingdom National Screening Committee (UKNSC), and the United States Preventive Services Task Force (USPSTF) as of 5 September 2016. Eligible recommendations focused on using self-report questionnaires to identify patients with presently experienced health problems or symptoms. Within each recommendation and accompanying evidence review we identified screening RCTs.

**Results:**

We identified 22 separate recommendations on questionnaire-based screening, including three CTFPHC recommendations against screening, eight UKNSC recommendations against screening, four USPSTF recommendations in favor of screening (alcohol misuse, adolescent depression, adult depression, intimate partner violence), and seven USPSTF recommendations that did not recommend for or against screening. In the four cases where the USPSTF recommended screening, either the CTFPHC, the UKNSC, or both recommended against. When recommendations diverged, the USPSTF expressed confidence in benefits based on indirect evidence, evaluated potential harms as minimal, and did not consider cost or resource use. CTFPHC and UKNSC recommendations against screening, on the other hand, focused on the lack of direct evidence of benefit and raised concerns about harms to patients and resource use. Of six RCTs that directly evaluated screening interventions, five did not report any statistically significant primary or secondary health outcomes in favor of screening, and one trial reported equivocal results.

**Conclusions:**

Only the USPSTF has made any recommendations for screening with questionnaires for presently experienced problems or symptoms. The CTFPHC and UKNSC recommended against screening in all of their recommendations. Differences in recommendations appear to reflect differences in willingness to assume benefit from indirect evidence and different approaches to assessing possible harms and resource consumption. There were no examples in any recommendations of RCTs with direct evidence of improved health outcomes.

## Background

Health screening involves the use of tests to identify apparently healthy people with early stage disease who do not have, or have not recognized that they have, symptoms or signs of the condition being screened. Screening is premised on the idea that early identification of asymptomatic pre-clinical disease can increase the likelihood of effective intervention and, thus, improve future health [1, 2]. Since the 1960s, when screening for breast cancer with mammography was first tested, enthusiasm for the idea that some diseases can be prevented through early detection has resulted in an explosion in the number of screening tests that have been promoted, some with evidence of benefit and others without such evidence [3].

This enthusiasm has also resulted in an expansion of the scope of screening itself. In addition to the goal of reducing risk of future ill health by detecting pre-clinical indicators of disease, the idea of screening has increasingly been applied to the use of self-report questionnaires to “screen” for existing health problems (e.g., alcohol misuse) or symptom-based syndromes (e.g., depression) that are not hidden; rather, they are experienced by patients, but not reported as health problems or observed by healthcare providers. The first example of a major national preventive care recommendation for this type of screening was the 2002 United States Preventive Services Task Force (USPSTF) recommendation for depression screening among adults in primary care [4]. Questionnaire-based screening has since been evaluated for other presently experienced health problems and symptom-based syndromes, including alcohol misuse, illicit substance use, intimate partner violence, and developmental delays in young children [5–7].

However, screening with questionnaires for existing conditions is controversial [8, 9], and major guideline organizations have reached different conclusions about the potential benefits versus harms of some of these programs [5–7]. Indeed, there are a number of reasons why applying a conventional test-based screening paradigm to presently experienced problems and symptoms may not improve health outcomes compared to providing patients with accurate healthcare information and appropriate assessment and intervention when problems are recognized. One such reason is that some of the conditions being screened may not necessarily be progressive. For some patients, symptoms and problems identified via self-report questionnaires reflect transitory reactions to circumstances that will resolve without intervention [8, 9]. Another is that using tests to identify and label medical conditions that patients do not otherwise recognize or report as health problems risks identifying large numbers of patients with mild conditions whose symptoms or problems may not be amenable to healthcare interventions. Finally, interventions to reduce symptoms or solve health problems are most effective when there is agreement between patients and providers on the impact of the problem and the need to address it. Such an agreement may not be present when tests are used to inform patients that they are experiencing a healthcare problem which they did not recognize as such [10].

Recommendations for screening should ideally be based on direct evidence from high-quality randomized controlled trials (RCTs) that show a sufficiently large benefit to justify the costs and harms involved in screening [1, 2, 10–12]. RCTs designed to directly test the effectiveness of a screening program should, at a minimum, (1) randomize patients prior to the screening intervention and (2) provide similar treatment resources to patients detected with the condition or health problem in the screening and non-screening arms of the trial so as not to confound the effects of a screening program with the effects of providing different treatments. Ideally, RCTs of screening programs would also exclude patients who are already known to have the targeted condition at the time of screening, as these patients would not be screened in actual practice [11].

The objective of the present study was to examine recommendations from three major national guideline organizations, the Canadian Task Force on Preventive Health Care (CTFPHC), the United Kingdom National Screening Committee (UKNSC), and the USPSTF, to (1) document the consistency of recommendations on using questionnaires to screen for presently experienced health problems or symptom-based syndromes, (2) identify sources of divergent recommendations, and (3) determine if guideline organizations have identified any examples of direct evidence from RCTs that questionnaire-based screening programs improve health outcomes for screened patients compared to non-screened patients.

## Methods

### Identification of eligible screening recommendations and data extraction

To identify eligible screening recommendations, we reviewed the most recent version of all guideline and recommendation statements listed on the websites of the CTFPHC [5], the UKNSC [6], and the USPSTF [7]. We considered only completed guideline and recommendation statements, but not “upcoming guidelines” or “recommendations in progress.” Eligible guidelines and recommendations were those that primarily focused on the use of a self-report questionnaire to identify patients with previously unreported and undetected yet presently experienced health problems or symptom-based syndromes. Guidelines and recommendations that focused on the use of performance-based measures, such as measures designed to test for cognitive impairment, but not self-report symptom questionnaires, were excluded.

The names of all guideline and recommendation statements listed on the websites of the CTFPHC, UKNSC, and USPSTF were uploaded into the systematic review data management program DistillerSR (Evidence Partners, Ottawa, Canada). DistillerSR was used to store and track results of the inclusion and exclusion process and for data extraction. When guideline and recommendation statements included more than one recommendation (e.g., one for children and one for adolescents), each recommendation was listed separately. For each included recommendation, we extracted the recommendation that was made (e.g., recommendation for screening, recommend against screening, insufficient evidence). Two investigators independently reviewed all recommendations to assess eligibility and extract the recommendations made. Any disagreements were resolved by consensus with a third investigator, if necessary.

### Sources of divergent recommendations

In cases where recommendations differed between guideline organizations, we extracted information on the main rationales provided for recommendations. One investigator initially extracted the rationales from the recommendation statements, and a second investigator validated the information extracted against the statements. Any disagreements were resolved by consensus, including a third investigator, if necessary. We compared rationales and identified where they diverged.

### Identification and evaluation of direct evidence from RCTs described in recommendations

We reviewed each recommendation statement and its accompanying evidence review and extracted the citations of all RCTs described as screening interventions; non-randomized interventions were excluded. If there were separate sections in the recommendation statement or evidence review for trials of screening interventions and for trials of treatment interventions, we extracted citations for all trials listed in the screening intervention section. If there were no separate sections, we extracted only citations for trials described as screening intervention trials. If the recommendation statement or evidence review described a systematic review of screening intervention trials, we extracted the citations for all eligible RCTs included in the systematic review.

In order to identify direct tests of screening interventions for each RCT that was described in a recommendation or accompanying evidence review as a screening trial, we determined (1) if patient eligibility and randomization occurred prior to administering the screening test and (2) if similar management resources were available to patients identified as having the target condition in both the screening and non-screening trial arms. Additionally, we determined if patients with a recent diagnosis of the target condition and patients being treated for the condition at the time of trial enrollment were excluded from the trial.

For included RCTs that directly evaluated screening interventions based on having (1) randomized patients prior to administering the screening test and (2) providing similar management resources to patients with the condition in the screening and non-screening trial arms, we extracted the primary and secondary health outcomes assessed in the RCT and determined if the outcomes were statistically significant or not. Process-based outcomes, such as the number of patients diagnosed or the number of patients who received treatment, were not extracted since these outcomes do not reflect improvements in health. If intent-to-treat and completer-only outcomes were provided, we extracted only intent-to-treat results. We did not extract subgroup outcomes, but only outcomes for main analyses that included all patients randomized to the screening and non-screening trial arms.

We determined if each screening trial had been registered, and, if so, we compared published outcomes to registered outcomes to identify any relevant discrepancies. If there was a pre-enrollment trial registration, and if published and registered outcomes differed, we recorded whether the trial outcome related to demonstrating benefit would have been different if pre-trial registered outcomes had been used. To identify whether trials had been registered, we first attempted to retrieve trial registration data, including the registration number, from each published article. If no registration information was included in the article, we searched for a trial registration in multiple clinical trial registries, including the ClinicalTrials.gov registry (www.ClinicalTrials.gov), the International Standard Randomized Controlled Trial Number registry (www.isrctn.com), the World Health Organization registry search portal (http://www.who.int/ictrp/search/en/), and the registry from the country of the first author (e.g., Netherlands Trial Register; www.trialregister.nl). To identify registry records, we performed a search using key terms from the published article, then attempted to match the principal investigator, funding source, intervention, control group, and design from the article to the registrations obtained in the search. If this method did not uncover a registration number, we contacted the corresponding author by email to attempt to determine if there was a trial registration that we had not been able to identify. Data were extracted by two investigators independently with any disagreements resolved through consultation with a third investigator.

## Results

### Recommendations on screening with self-report questionnaires

**Fig. 1:**
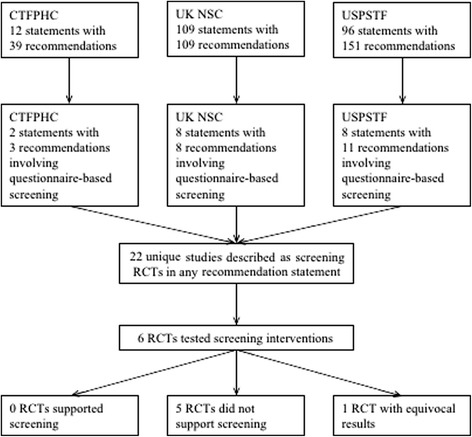
Flow of guideline and recommendation statements reviewed and included, randomized controlled trials described in the statements, and results of randomized controlled trials that were tests of questionnaire-based screening interventions

As of 5 April 2016, there were 217 guideline or recommendation statements with 299 separate recommendations posted on the websites of the CTFPHC (12 statements with 39 recommendations), UKNSC (109 statements with 109 recommendations), and USPSTF (96 statements with 151 recommendations). Of these, there were 18 guideline or recommendation statements with 22 separate recommendations that focused on questionnaire-based screening, including two statements with three recommendations from the CTFPHC, eight statements with eight recommendations from the UKNSC, and eight statements with 11 recommendations from the USPSTF. No additional recommendations related to questionnaire-based screening were identified when the websites were reviewed again on 5 September 2016 (Fig. 1).

As shown in Table 1, the CTFPHC made two weak recommendations and one strong recommendation against screening. The UKNSC recommended against screening in all eight of its recommendations. The USPSTF, on the other hand, made four recommendations to offer screening and determined in seven cases that there was insufficient evidence to recommend for or against screening. In conditions where more than one organization made a recommendation for or against screening in the same patient population, the USPSTF recommended using questionnaires to screen for alcohol misuse, but the UKNSC recommended against it; the USPSTF recommended screening adults, including women in pregnancy and postpartum for depression, whereas the CTFPHC and UKNSC recommended against; both the CTFPHC and the UKNSC recommended against screening for developmental delays or behavioral problems; and the USPSTF recommended screening for intimate partner violence, whereas the UKNSC recommended against.

**Table 1:** Characteristics of CTFPHC, UKNSC, and USPSTF guidelines that provide recommendations for questionnaire-based screening

Guideline Organization Year	Condition	Target population for recommendation	Recommendation
Alcohol Misuse and Illicit Drug Use			
UKNSC 2011	Alcohol misuse	Adults	Screening program not recommended
USPSTF 2013	Alcohol misuse	Adults	Recommends offering the service (B Grade)
USPSTF 2013	Alcohol misuse	Adolescents aged 12–17 years	Insufficient evidence to assess balance of benefits and harms (I Grade)
USPSTF 2008	Illicit drug use	Adults	Insufficient evidence to assess balance of benefits and harms (I Grade)
Depression and Psychiatric Illness			
CTFPHC 2013	Depression	Adults not at increased risk	Weak recommendation against
CTFPHC 2013	Depression	Adults who may be at increased risk	Weak recommendation against
UKNSC 2011	Depression	Women in postnatal period	Screening program not recommended
UKNSC 2015	Depression	Adults	Screening program not recommended
USPSTF 2016	Depression	Adults, including pregnant and postpartum women	Recommends offering the service (B Grade)
USPSTF 2016	Depression	Adolescents aged 12–17 years	Recommends offering the service (B Grade)
USPSTF 2016	Depression	Children aged 11 years or younger	Insufficient evidence to assess balance of benefits and harms (I Grade)
UKNSC 2006	Psychiatric illness	Adults	Screening program not recommended
Developmental Delay, Behavioral Problems, and Speech and Language Delay, Autism			
UKNSC 2005	Developmental and behavioral problems	Children	Screening program not recommended
CTFPHC 2016	Developmental delay	Children aged 1–4 years	Strong recommendation against
USPSTF 2015	Speech and language delay and disorders	Children aged 5 years or younger	Insufficient evidence to assess balance of benefits and harms (I Grade)
UKNSC 2012	Autism spectrum disorder	Children below age 5	Screening program not recommended
USPSTF 2016	Autism spectrum disorder	Children aged 18–30 months	Insufficient evidence to assess balance of benefits and harms (I Grade)
Domestic Violence, Intimate Partner Violence, and Abuse of Elderly and Vulnerable Adults			
UKNSC 2006	Domestic violence	Pregnant women	Screening program not recommended
UKNSC 2013	Domestic violence	Adult women	Screening program not recommended
USPSTF 2013	Intimate partner violence	Adult women of childbearing age	Recommends offering the service (B Grade)
USPSTF 2013	Abuse and neglect of elderly and vulnerable adults	Elderly and vulnerable adults (physical or mental)	Insufficient evidence to assess balance of benefits and harms (I Grade)
Suicide Risk			
USPSTF 2014	Suicide risk	Adolescents, adults, and older adults without an identified psychiatric disorder	Insufficient evidence to assess balance of benefits and harms (I Grade)

*CTFPHC* Canadian Task Force on Preventive Healthcare, *UKNSC* United Kingdom National Screening Committee, *USPSTF* United States Preventive Services Task Force

### Sources of divergent recommendations

We compared divergent recommendations for versus against screening, but did not consider “I” recommendations by the USPSTF in our assessment of divergent recommendations. As shown in Table 2, USPSTF recommendation statements in favor of screening for alcohol misuse in adults, depression screening of adolescents, and intimate partner violence in adult women all recognized that there was no direct RCT evidence of benefit from screening. Instead, the USPSTF expressed confidence that screening would result in benefit based on indirect evidence from studies of screening test accuracy and intervention effectiveness. The CTFPHC and UKNSC, on the other hand, emphasized the lack of direct trial evidence of effectiveness in their recommendations against screening.

**Table 2:** Comparison of main rationales provided for recommendations for and against screening

Condition	Recommendation(s) in favor of screening	Rationale for recommending screening	Recommendation(s) against screening	Rationale for recommending against screening
Alcohol misuse	USPSTF 2013	• Recognized lack of direct evidence from randomized controlled trials of screening interventions • Reported adequate evidence for screening test accuracy and behavioral interventions to reduce alcohol misuse • Indicated that harms likely small to none	UKNSC 2011	• Emphasized limited overall evidence and no evidence of improved health outcomes from randomized controlled trials of screening programs
Depression	USPSTF 2016 (Adults)	• Reported that there was adequate evidence that programs that combined screening and support improved clinical outcomes • Reported adequate evidence for screening test accuracy and depression treatments • Indicated that there was adequate evidence that harms of screening are small to none	CTFPHC 2013 (Adults)	• Emphasized lack of evidence from randomized controlled trials of screening programs • Specifically indicated that the systematic review for the USPSTF guidelines conflated screening and treatment • Raised concern about harms of potentially high rate of false positive screens, about the applicability of treatment evidence to screened patients, and about resource implications in absence of evidence of benefit
	USPSTF 2016 (Adolescents)	• Recognized lack of direct evidence from randomized controlled trials of screening interventions • Reported adequate evidence for screening test accuracy and depression treatments • Indicated that screening is unlikely to be associated with significant harms and harms would be small for pharmacological treatments if properly monitored	UKNSC 2011 (Postnatal women)	• Emphasized lack of evidence from randomized controlled trials of improved maternal or infant outcomes from depression screening • Noted lack of evidence of cost-effectiveness
			UKNSC 2015 (Adults)	• Emphasized lack of evidence from randomized controlled trials of improved health outcomes • Raised concern about harms due to false positive screens and overtreatment and about cost-effectiveness
Domestic or intimate partner violence	USPSTF 2013 (Adult women)	• Recognized lack of direct evidence from randomized controlled trials of screening interventions • Reported adequate evidence for screening test accuracy and interventions to reduce harms from violence • Indicated that harms “no greater than small”	UKNSC 2006 (Pregnant women)	• No report available
			UKNSC 2013 (Adult women)	• Emphasized lack of evidence of improved health outcomes from randomized controlled trials of screening programs

In the case of adult depression screening, the USPSTF argued that there was direct trial evidence of benefit of combined screening and management support. The UKNSC indicated that there were no trials that had shown direct evidence of effectiveness of screening. The CTFPHC similarly indicated that there was no direct trial evidence of the benefit of screening programs. In the CTFPHC recommendation, it was specifically noted that the trials identified in the systematic review performed in conjunction with the USPSTF recommendation conflated screening and enhanced collaborative depression care and that it was not necessarily the case that screening was a necessary component.

Another key difference between organizations was related to the treatment of resource utilization and possible harms from screening. The USPSTF does not consider costs in their recommendations, and in each of their recommendations in favor of screening, they indicated that any harms would be small to negligible. The CTFPHC and UKNSC, on the other hand, did raise concerns about resource consumption in the absence of evidence of benefit and about harms to patients who would be screened, including overdiagnosis and overtreatment.

### Evaluation of direct RCT evidence on screening interventions described in recommendations

As shown in Fig. 1, there were 22 unique RCTs that were described in the recommendation statements or accompanying evidence reviews (see Table 3 for trial characteristics). Of these, only six met the two criteria for being a direct test of a screening intervention; that is, they randomized patients prior to administering the screening questionnaire and provided similar resources for management of patients identified as needing care in the screening and non-screening trial arms [13–19]. Of the other 16 trials, 10 included questionnaire scores as part of trial eligibility criteria, but they were trials that evaluated a specific treatment compared to usual care for people identified with the condition of interest, not whether screening would benefit patients compared to not screening [20–30]. The other RCTs randomized patients post-screening [31] or screened post-randomization, but provided superior care options to patients identified in the screening arm compared to patients identified as needing care in the non-screening arm [32–36].

**Table 3:** Characteristics of randomized controlled trials described in CTFPHC, UKNSC, and USPSTF guidelines

Condition/guideline	First author Year Country	Number of patients randomized	Eligibility and randomization	Determined eligibility and randomized patients prior to screening?	Diagnostic/treatment status	Excluded already diagnosed and already treated patients?	Management	Similar management options for screened and unscreened trial arms?
Alcohol Misuse								
UKNSC	Fleming 1997 USA [20]	774	Adult primary care practice patients who screened positive for problem drinking (men > 14 drinks per week, women > 11 drinks per week) were eligible and randomized	No	Patients with alcohol treatment program in the previous year or advice from physician to change alcohol use in previous 3 months were excluded	Yes	Intervention arm: general health booklet, 15-minute brief intervention and reinforcement session 1 month later; Control arm: general health booklet only	No
	Wutzke 2002 Australia [21]	554	Adult general practice patients who screened positive for problem drinking (men ≥ 300 g weekly, women ≥ 180 g weekly; two or more episodes of intoxication a month; or experiencing alcohol-related harm in the previous 6 months) were eligible and randomized	No	Patients with a history of hospital admission for an alcohol-related disorder or who had received advice from a health professional to abstain from alcohol were excluded	Yes	Intervention arm 1: 5 minutes of brief advice and a leaflet plus 15 minutes of counseling Intervention arm 2: 15 minutes of counseling with 2 additional counseling sessions; Control arm: 5 minutes of brief advice and a leaflet	No
	Crawford 2004 UK [22]	599	Adult emergency department patients who screened positive for alcohol misuse (men > 8 units of alcohol in any one session at least once a week, women > 6 units of alcohol in any one session at least once a week; patient believed their attendance in the emergency department could be related to alcohol) were eligible and randomized	No	Patients already in contact with alcohol services or who requested help with alcohol problems were excluded	Yes	Intervention arm: patients given an informational leaflet and offered a follow-up appointment with an alcohol health worker; Control arm: informational leaflet only	No
	Beich 2007 Denmark [23]	906	Adult primary care patients who screened positive on the AUDIT (score > 8 and < 21) with a maximum weekly consumption of 35 drinks were eligible and randomized	No	Patients receiving treatment for an alcohol use disorder at the time of enrollment were excluded	Yes	Intervention arm: patients offered feedback on present drinking, advice on reducing drinking, a self-help booklet, and an invitation for follow-up consultation; Control arm: no intervention received	No
Depression								
USPSTF	Callahan 1994 USA [24]	175	Adult primary care patients with CES-D ≥ 16 and HAMD ≥ 15 were eligible and randomized	No	21% of enrolled patients already diagnosed and 12% already on antidepressants (overlap not specified)	No	Intervention arm: enhanced depression care; Control arm: usual care	No
	Williams 1999 USA [13]	969^a^	Adult medical patients eligible and randomized to (1) screening with single mood question, (2) screening with the CES-D, or (3) usual care; depression outcomes only assessed for 97 patients with major depression at baseline and a random sample of 119 other patients	Yes^a^	Only 11 of 41 physician diagnoses of depression were new diagnoses (27%); patients classified as new diagnoses if no evidence of diagnosis in chart and patient reported that not diagnosed or treated in last 2 years^b^	No	Both groups received usual care	Yes
	Wells 2004 USA [25]	1356	Primary care clinics randomized; adult patients with probable depressive disorder were eligible	No	In the 6 months prior to trial, 48% of patients discussed emotional issues at medical visit; 29% had specialty mental health visit; 44% getting appropriate mental health care	No	Intervention arm: enhanced depression care; Control arm: usual care	No
	Whooley 2000 USA [32]	2346^c^	Primary care patients ≥ 65 years were eligible and randomized to screening with GDS and seven educational sessions versus usual care; only 331 patients with GDS ≥ 6 at baseline were included in depression outcome analysis	Yes^c^	In the 12 months prior to trial, 20% of patients in outcome analysis prescribed antidepressant medication	No	Intervention arm: patients offered 6 weekly educational sessions on depression plus 1 booster session; Control arm: usual care	No
	Rost 2001 USA [26]	479	Primary care practices randomized; adult patients with five or more symptoms of current major depressive disorder were eligible	No	In the 6 months prior to trial, 44% of patients were prescribed antidepressant medication or had a specialty mental health care visit	No	Intervention arm: enhanced depression care; Control arm: usual care	No
	MacArthur 2002 UK [33]	2064	Midwife practices randomized; women receiving postnatal care in participating midwife practices were eligible Midwives used a symptom checklist and the EPDS to inform care plans and visit scheduling in intervention group, but not for screening	Yes	Existing depression diagnosis or treatment not in exclusion criteria; no information on depression diagnosis or treatment provided at time of enrollment	No	Intervention arm: multifaceted care enhancement, including training of midwives to implement new model of care and use of symptom checklist; Control arm: usual care	No
	Jarjoura 2004 USA [27]	61	Adult internal medicine patients positive for depression on PRIME-MD were eligible and randomized	No	Patients receiving intervention for mental health problems or seeking help for depression or other emotional problems were excluded	Yes	Intervention arm: nurse-supported depression management and referral program; Control arm: usual care	No
	Bergus 2005 USA [31]	51	Adult family practice patients with low mood or anhedonia in last 2 weeks based on PHQ-9 were eligible and randomized to have their PHQ-9 scores disclosed or not to their physician	No	38% of enrolled patients on medication for depression or anxiety at time of enrollment and 60% had a history of depression treatment	No	Both groups received usual care	Yes
	Bijl 2003 [28] Bosmans 2006 Netherlands [29]	145	General practices randomized; patients with GDS ≥ 5 and positive for depression on PRIME-MD were eligible	No	Patients using antidepressants at time of trial enrollment were excluded	Yes	Intervention arm: enhanced depression care; Control arm: usual care	No
	Morrell 2009 UK [34]	4084	General practices randomized; only women with EPDS ≥ 12 at 6 weeks postpartum were included in outcome analyses	Yes^d^	Excluded women with severe mental health problems, but existing depression diagnosis or treatment not part of exclusion criteria; no information on depression diagnosis or treatment provided at time of enrollment	No	Intervention arm: home visits from health visitors with training in psychological approaches, along with screening and psychological interventions; Control arm: usual care	No
	Leung 2011 Hong Kong [14]	462	Women attending maternal and child health centers for routine child health services were eligible and randomized to screening with the EPDS versus usual care	Yes	Patients receiving psychiatric treatment were excluded	Yes	Both groups eligible to receive nurse counseling or a community psychiatry referral	Yes
	van der Weele 2012 Netherlands [30]	239	Primary care practices randomized; patients ≥ 75 years with GDS-15 scores ≥ 5 were eligible	No	Patients receiving treatment for depression at the time of enrollment were excluded	Yes	Intervention arm: stepped-care consisting of individual counseling, coping with depression course, and possible referral to general practitioner to discuss further treatment; Control arm: usual care	No
	Yawn 2012 USA [35]	2343^d^	Primary care practices were randomized to a complex depression care intervention, including screening with EPDS and PHQ-9, versus usual care; women 5 to 12 weeks postpartum were eligible; only 408 patients with positive depression screen at baseline were included in depression outcome analysis	Yes^e^	Existing depression diagnosis or treatment not in exclusion criteria; no information on depression diagnosis or treatment provided at time of enrollment	No	Intervention arm: enhanced depression care; Control arm: usual care	No
Developmental Delay (CTFPHC) and Speech and Language Delay (USPSTF)								
CTFPHC	Guevara 2013 USA [15]	2103	Pediatric patients < 30 months old, > 36 weeks’ estimated gestational age, with no major congenital anomalies or genetic syndromes, not in home foster care, and not currently receiving early intervention services were eligible and randomized to (1) screening with office assistance, (2) screening without office assistance, or (3) standard developmental surveillance without screening	Yes	Children receiving early intervention at the time of enrollment were excluded	Yes	Both groups eligible for referrals to early intervention services	Yes
CTFPHC USPSTF	de Koning 2004 [16] van Agt 2007 Netherlands [17]	10,355	Child healthcare physicians randomized to screening with the VTO Language Screening Instrument versus standard developmental surveillance without screening; children aged 15 to 18 months were eligible	Yes	Existing developmental delay diagnosis or treatment not in exclusion criteria; no information on developmental delay diagnosis or treatment provided at time of enrollment	No	Both groups eligible for standard speech and language assessments and early intervention	Yes
Domestic Violence (UKNSC) and Intimate Partner Violence (USPSTF)								
UKNSC USPSTF	MacMillan 2009 Canada [18]	6743	Female primary care, emergency department, or obstetrics/gynecology patients who had a male partner at some point in the last 12 months were eligible and randomized to be screened with the WAST versus usual care	Yes	Already receiving treatment or help for domestic violence not in exclusion criteria; no information provided on how many women were receiving help for partner violence at time of enrollment	No	Both groups eligible to receive an information card with contact details for locally available resources for women exposed to violence	Yes
UKNSC	Klevens 2012 USA [36]	2708	Female primary care patients eligible and randomized to (1) screening with the Partner Violence Screen instrument, (2) no screening (all receive a partner violence resource list), or (3) no screening (no list)	Yes	Already receiving treatment or help for domestic violence not in exclusion criteria; no information provided on how many women were receiving help for partner violence at time of enrollment	No	Intervention arm 1: women with positive screens receive informational video about hospital-based partner advocacy program, plus a partner violence resources list and a general resource list; Women with negative screen receive general resources list only; Intervention arm 2: partner violence resource list and general resource list to all women; Control arm: general resource list only to all women	No
Suicide Risk								
USPSTF	Crawford 2011 UK [19]	443	Adult primary care patients with signs of depression (“yes” to two-item screener) were eligible and randomized to be screened or not for suicide risk	Yes	Already receiving treatment or help for suicide risk not in exclusion criteria; no information provided on how many patients receiving treatment at time of enrollment	No	Intervention arm: patients with positive screens encouraged to use resources already available to them; Control arm: not described, but assumed to be same usual care as in intervention arm	Yes

^a^Eligibility was determined and randomization occurred pre-screening; however, only 216 of 969 patients randomized (23%) were assessed for depression outcomes

^b^Based on published article and clarification provided by corresponding author

^c^Eligibility was determined and randomization occurred pre-screening; however, only 331 of 2346 patients randomized (14%) were included in depression outcome analysis

^d^Practices were randomized pre-screening; however, only 418 patients with EPDS scores of at least 12 were included in depression outcome analyses

^e^Eligibility was determined and randomization occurred pre-screening; however, of the 2343 patients randomized, only 408 (17%) with positive depression screens on the EPDS or PHQ were assessed for depression outcomes

*AUDIT* Alcohol Use Disorder Identification Test, *CES*-*D* Center for Epidemiologic Studies Depression Scale, *CTFPHC* Canadian Task Force on Preventive Healthcare, *HAM*-*D* Hamilton Rating Scale for Depression, *EPDS* Edinburgh Postnatal Depression Scale, *GDS* Geriatric Depression Scale, *PHQ*-*9* Patient Health Questionnaire-9, *PRIME*-*MD* Primary Care Evaluation of Mental Disorders, *UKNSC* United Kingdom National Screening Committee, *USPSTF* United States Preventive Service Task Force, *VTO* VroegTijdige Onderkenning Ontwikkelingsstoornissen, *WAST* Women Abuse Screening Tool

As shown in Table 4, of the six RCTs that directly tested screening interventions, two tested depression screening interventions [13, 14], two tested interventions for screening for developmental or speech and language delays [15–17], one tested an intimate partner violence screening intervention [18], and one tested a suicide risk screening intervention [19]. In five of the RCTs [13, 15–19], no primary or secondary health outcomes were statistically significant in favor of the screening intervention. In the other RCT [14], a trial of depression screening in postpartum women from Hong Kong, of the two primary outcomes that were registered, one generated statistically significant results, whereas the other did not. The published trial report, however, only identified the statistically significant outcome as primary and relegated the non-significant outcome to secondary.

**Table 4:** Primary and secondary health outcomes reported in randomized controlled trials that (1) determined eligibility and randomized patients prior to screening and (2) provided similar management options for screened and unscreened trial arms

First author Year Country	Trial registration number	Trial assessed health outcomes?^a^	Primary health outcome(s)^a,b^	Primary health outcomes statistically significant in favor of screening intervention?	Other health outcome(s)^b,c^	Other health outcomes statistically significant in favor of screening intervention?
Alcohol Misuse						
No randomized controlled trials of screening interventions						
Depression						
Williams 1999 USA [13]	Not registered	Yes	1. Prevalence of depression assessed by the DIS at 3 months^d^	1. No	None^d^	Not applicable
Leung 2011 Hong Kong [14]	NCT00251342	Yes	1. Depressive symptoms (dichotomous) measured by the EPDS at 6 months (Published)^e^ 2. Depressive symptoms (continuous) measured by the EPDS at 6 months (Registered)^e^ 3. Mental health symptoms (continuous) measured by the GHQ-12 at 6 months (Registered)^e^	1. Yes 2. Yes 3. No	1. Parental stress measured by the PSI at 6 and 18 months 2. Parental stress measured by the PSI-PD at 6 and 18 months 3. Experiencing difficult parent-child interaction measured by the PSI-PCDI at 6 and 18 months 4. Experiencing difficult child measured by the PSI-DC at 6 and 18 months 5. Marital satisfaction measured by the CKMSS score at 6 and 18 months 6. Depressive symptoms (dichotomous) measured by the EPDS at 18 months 7. Depressive symptoms (continuous) measured by the EPDS at 18 months 8. Mental health symptoms measured by the GHQ-12 at 18 months	1. No, No 2. No, No 3. No, No 4. No, No 5. No, No 6. No 7. No 8. No
Developmental Delay (CTFPHC) and Speech and Language Delay (USPSTF)						
Guevara 2013 USA [15]	NCT00844246	No^f^	Not applicable	Not applicable	Not applicable	Not applicable
de Koning 2004 [16] van Agt 2007 Netherlands [17]	Not registered	Yes	1. Need special education at age 8^g^ 2. Has repeated a grade by age 8^g^ 3. Has repeated a grade due to language problems by age 8^g^ 4. Below 10th percentile on grade 2 oral language tests^g^ 5. Below 10th percentile on grade 2 reading tests^g^ 6. Below 10th percentile on grade 2 spelling tests^g^ 7. Teacher predicts normal development in future^g^	1. No 2. No 3. No 4. No 5. No 6. No 7. No	1. Language comprehension measured by the VTO Language Screening Instrument at 36 months^g^ 2. Language production measured by the van Wiechen items plus VTO Language Screening Instrument PQ scores at 36 months^g^	1. No^h^ 2. No^h^
Domestic Violence (UKNSC) and Intimate Partner Violence (USPSTF)						
MacMillan 2009 Canada [18]	NCT00182468	Yes	1. Recurrence of intimate partner violence measured with the CAS at 6, 12, and 18 months 2. Quality of life measured with the WHOQOL-Brief at 6, 12, and 18 months	1. No, No, No 2. No, No, No	1. Depressive symptoms measured by the CES-D at 6, 12, and 18 months 2. PTSD symptoms as measured by the SPAN at 6, 12, and 18 months 3. Alcohol abuse/dependency as measured by the TWEAK at 6, 12, and 18 months 4. Drug abuse measured by the DAST at 6, 12, and 18 months 5. Global health and well-being as measured by the SF-12 at 6, 12, and 18 months	1. No, No, No 2. No, No, No 3. No, No, No 4. No, No, No 5. No, No, No
Suicide Risk						
Crawford 2011 UK [19]	ISRCTN84692657	Yes	1. Thoughts that life not worth living 10–14 days post-randomization	1. No	1. Wishing to be dead 2. Thoughts of committing suicide	1. No 2. No

^a^Health outcomes are outcomes that reflect patient-experienced health and well-being; receipt of healthcare services is not included

^b^Intent-to-treat results used if both intent-to-screen and non-intent-to-screen analyses published

^c^Secondary health outcomes do not include subgroup analyses of a subset of patients included in main analyses

^d^All additional outcomes were based on subgroups of patients in main analysis

^e^Primary outcome per trial registration was “Mother's mental health at 6 months postpartum, as measured on the EPDS and GHQ-12” without specifying method of aggregation; publication described dichotomous EPDS as the primary outcome and continuous EPDS and GHQ-12 scores as secondary outcomes

^f^Outcomes include only diagnoses and referrals and time to diagnosis and referral, but no health outcomes

^g^Primary outcomes published in van Agt et al. [17] and secondary outcomes in de Koning et al. [16]

^h^Per Table 2 in de Koning et al. [16], mean language and production scores higher for control than intervention group; all other 36-month outcomes related to diagnoses and services

*CAS* Composite Abuse Scale, *CES*-*D* Center for Epidemiologic Studies Depression Scale, *CKMSS* Chinese Kansas Marital Satisfaction Scale, *DAST* Drug Abuse Severity Test, *DIS* Diagnostic Interview Schedule, *EPDS* Edinburgh Postnatal Depression Scale, *GHQ*-*12* General Health Questionnaire –12, *PSI* Parenting Stress Index, *PSI*-*DC* Parenting Stress Index – Difficult Child, *PSI*-*PCDI* Parenting Stress Index – Parent-Child Dysfunctional Interaction, *PSI*-*PD* Parenting Stress Index – Parental Distress, *PTSD* posttraumatic stress disorder, *PQ* Parent Questionnaire, *SF*-*12* Short Form – 12, *SPAN* Startle, Physiological Arousal, Anger, and Numbness, *TWEAK* “tolerance, worry, eye-opener, amnesia, cut down”, *VTO* VroegTijdige Onderkenning Ontwikkelingsstoornissen, *WHOQOL*-*Brief* World Health Organization Quality of Life – Brief

## Discussion

Screening for presently experienced health problems and symptom-based syndromes with self-report questionnaires has been evaluated by the CTFPHC, UKNSC, or USPSTF in the areas of alcohol misuse, depression, developmental or speech and language delays, domestic violence, and suicide risk. The CTFPHC and UKNSC have made a total of 11 recommendations against screening with self-report questionnaires and no recommendations in favor of the practice. The USPSTF, on the other hand, has made four recommendations in favor of questionnaire-based screening programs (alcohol misuse, adult depression, adolescent depression, intimate partner violence) and no recommendations against screening. In seven other cases, the USPSTF determined that there was insufficient evidence to recommend for or against the service (“I” recommendation).

The CTFPHC, UKNSC, and USPSTF all attempt to evaluate the balance between possible benefits and possible harms that would be accrued from screening programs. The methods the groups use are generally similar, although there are some differences. Both the CTFPHC and USPSTF include methods for evaluating screening pathways based on indirect evidence, such as evidence on screening test accuracy and treatment effectiveness [37, 38]. They differ, however, in that the CTFPHC uses the GRADE system [39] and makes weak or strong recommendations for or against all preventive care services it evaluates; the USPSTF, on the other hand, uses its own rating system and may make an “I” recommendation, which reflects that its members do not believe that there is sufficient evidence to make any recommendation. The UKNSC differs from both the CTFPHC and USPSTF in that it uses a list of criteria, including the availability of evidence from high-quality RCTs, to evaluate screening programs [10]. In addition, the CTFPHC and UKNSC, but not the USPSTF, consider resource use in their recommendations [10, 37, 38].

Divergences in recommendations between the USPSTF and the CTFPHC and UKNSC appear to stem from several sources. First, when recommendations diverge, the USPSTF has indicated in each case that there is at least moderate certainty that there would be at least moderate net benefit based on indirect evidence from studies of test accuracy and treatment of screen-detected symptomatic patients and, if available, potential harms of screening and treatment. The CTFPHC and UKNSC, on the other hand, have determined that those links are insufficient to establish that benefit would occur. Additionally, in the case of depression screening, the CTFPHC noted that the USPSTF relied upon RCTs of depression care management programs, which used screening tools to establish trial eligibility prior to randomization, as evidence on screening. Consistent with this, of the 13 RCTs described by the USPSTF as screening trials, only two randomized patients prior to screening and provided similar care options in patients with depression in the screen and no-screen trial arms (Table 3). Second, in divergent recommendations, the CTFPHC and UKNSC raised concerns about possible harms from screening, including overdiagnosis and overtreatment, whereas the USPSTF rated described harms as small to negligible in all recommendations in favor of screening and did not mention the possibility of overdiagnosis or overtreatment in any. Finally, cost and resource considerations were included in CTFPHC and UKNSC recommendations, but not in USPSTF recommendations.

No examples of direct RCT evidence that questionnaire-based screening improves health outcomes were described in the recommendations of the CTFPHC, UKNSC, or USPSTF. There were only six RCTs that directly tested screening interventions by randomizing patients prior to administering the screening questionnaire and providing similar management resources for patients identified as needing care in the screening and non-screening arms of the trials. In five of the trials, which evaluated whether screening for depression, developmental or speech and language delays, intimate partner violence, and suicide risk improved health compared to usual care, there were no statistically significant primary or secondary health outcomes in favor of the screening intervention.

In the sixth RCT, which tested depression screening among postpartum women in Hong Kong [14], based on outcome definitions registered prior to conducting the trial, there was one primary outcome that was statistically significant in favor of screening and one that was not. However, in the published outcome report, only the statistically significant outcome was described as a primary outcome; the non-statistically significant outcome was described as secondary [14]. As described previously [40, 41], there is concern that results from this trial may not represent what would likely occur in practice. In addition to reclassifying trial outcomes post hoc in a way that portrayed trial results as positive, rather than equivocal, the reported effect size was implausibly large. The authors randomized 231 women to be screened, of whom 55 received the low-intensity counseling treatment that was provided; 11 of 231 women in the control arm also received the treatment. The authors reported a standardized mean difference (SMD) effect size per woman screened on the Edinburgh Postnatal Depression Scale of 0.34, roughly equivalent to SMD = 1.81 for the 44 additional patients treated in the screened group compared to the control group. This reported effect per woman treated, however, is six to seven times the size of effects that are typically achieved with similar interventions in primary care settings [40, 41]. A meta-analysis of collaborative depression care treatment, for instance, reported an effect size of 0.25 SMD (N = 30 trials) [42]. Another meta-analysis of psychological treatment for adult depression in primary care reported an overall SMD effect size of 0.31 (N = 15 trials) [43]. None of the individual RCTs included in either meta-analysis approached the effect size reported per patient treated in the Hong Kong screening trial. Consistent with concerns that results from the Hong Kong trial may not be reproduced in actual practice, the only other trial of depression screening included in the present review did not find that depression screening significantly reduced the number of depression diagnoses among patients screened compared to patients not screened [13].

The USPSTF was recently criticized for relying upon indirect evidence and for not adequately considering potential harms in recommending depression screening [44]. Experts pointed out that there are numerous examples where the use of insufficient and indirect evidence has led to ineffective and harmful screening programs and argued that guideline makers should refrain from recommending new screening services based on only indirect evidence [44]. In the context of questionnaire-based screening programs, this concern is heightened because, when RCTs have directly tested these programs, they have not found evidence of health benefits. When high-quality trials are feasibly conducted, as is the case with questionnaire-based screening programs, a more conservative approach than recommending a new service without direct evidence would be to call for well-conducted RCTs.

Appropriate care that addresses patient needs, but avoids intervention without demonstrated benefit, is increasingly emphasized in healthcare planning and service delivery [45, 46]. Recognition that screening is not benign is reflected in recent recommendations for more restricted use of screening for breast [47, 48] and prostate cancer [49, 50]. Using self-report questionnaires as screening tests to identify unreported and unrecognized, but presently experienced, health problems and symptoms extends the boundaries of the standard screening paradigm, in which tests are used to detect hidden signs or unrecognized symptoms in order to stave off future health problems. It is possible that questionnaire-based screening might improve upon good, conscientious medical care that provides patients with information and encourages them to inquire about problems they are experiencing. Direct evidence from existing studies included in CTFPHC, UKNSC, and USPSTF recommendations, however, does not lead to this conclusion.

Without evidence that using questionnaires to search for presently experienced, unreported problems would lead to better health outcomes, the negative implications of this practice need to be carefully considered in screening recommendations, including the possibility that it would lead to overdiagnosis and overtreatment [51–54]. Traditionally, overdiagnosis has been understood to occur when a person without symptoms is diagnosed with a condition or disease that will not lead to symptoms or early mortality and would not ever be identified without screening [51, 52]. More broadly, in the case of presently experienced problems or symptoms, overdiagnosis can occur when patients are identified with a disorder or problem that they do not experience as significantly impairing and that would not be expected to be substantively affected by medical intervention [53, 54]. This could occur in mental disorders, even when diagnostic criteria are met, such as in the presence of mild depressive symptoms that fall close to the normal range on a diagnostic spectrum [54].

Potential harms have not been well documented in questionnaire-based screening, but if screening is done, some patients who would not otherwise be exposed will experience harms. For example, individuals may be exposed to unnecessary and ineffective treatments, undesirable medication effects, the labeling of problems that may resolve on their own as medical problems, and nocebo effects from telling patients who are not otherwise specifically concerned that they have a medical problem, such as depression [10, 55].

In addition to direct harms to patients, the practice would consume scarce healthcare resources that might be better devoted to providing services to patients who clearly have health problems, including mental health problems, but who in many cases receive less than adequate care [10, 56]. Some have argued that screening with questionnaires can be done at very little cost [57], and having patients respond to questionnaires is not typically expensive. However, screening involves much more than this, including follow-up assessments to separate true from false positives, consultations to determine the best management options, and treatment and follow-up services. One study found that, when depression screening is conducted, more than 70% of visits last more than 15 minutes and 17% last more than 30 minutes compared to 42% and 6%, respectively, when screening is not done, and this only factors in the time involved in the initial screening visit, but not follow-ups and referral management, for instance [58]. The number of patients who would follow this pathway depends on the clinical setting and condition targeted. In depression, 30% or more of patients in many settings would have positive screens and would need to be evaluated, even though most of these patients would not have depression [59, 60].

By 1996, based on a conservative estimate, a typical primary care physician needed to spend 7.4 hours per day just to minimally comply with Grade A and B recommendations (moderate to high certainty of moderate or high benefit, should be offered) for preventive care from the USPSTF [61]. Since then, the number of A and B recommendations has grown, including the recommendations for questionnaire-based screening described in the present study. Physicians cannot realistically comply with all USPSTF A and B recommendations, but guidance on how to prioritize is not provided. As a result, they may determine which recommendations to offer based on their own estimation of likely benefit and harm, as well as resources required. In depression screening, a national survey found that only 4% of American primary care patients were screened for depression in 2012–2013, even though it was recommended by the USPSTF and covered by the Affordable Care Act as of 2010 [62].

There are limitations to consider in evaluating the results of the present study. First, we included only recommendations from three guideline organizations, the CTFPHC, UKNSC, and USPSTF. Although these organizations are recognized for their leadership in the area of preventive healthcare policy, these results do not necessarily apply to other organizations that make recommendations on screening. Second, we only reviewed trials included in recommendation statements and did not seek to identify other trials that may have been conducted. It is possible that there are trials of questionnaire-based screening that we did not review from other areas of screening where no recommendations have been made or from trials conducted since these recommendations were made. However, identification of any existing trials was not the objective of the present study. Rather, we sought to determine if the CTFPHC, UKNSC, or USPSTF had identified direct evidence from any questionnaire-based screening program that would support the use of indirect evidence in recommendations.

## Conclusions

In summary, neither the CTFPHC nor the UKNSC has made any recommendations endorsing questionnaire-based screening. The USPSTF, on the other hand, has recommended questionnaire-based screening for alcohol misuse, depression in adolescents and adults, and intimate partner violence. Compared to the CTFPHC and UKNSC, the USPSTF appears to be more confident in relying upon indirect evidence, minimizes potential harms, and does not consider cost and resource utilization.

## Abbreviations

CTFPHC: Canadian Task Force on Preventive Health Care
RCT: randomized controlled trial
SMD: standardized mean difference
UKNSC: United Kingdom National Screening Committee
USPSTF: United States Preventive Services Task Force
